# Supplementary material for: Risk Factors and Clinical Characteristics of Acute Kidney Injury in Patients with COVID-19: A Systematic Review and Meta-Analysis
Source: Pathophysiology. 2023 May 15;30(2):233–47. doi: 10.3390/pathophysiology30020020 (PMC10204466; doi:10.3390/pathophysiology30020020)
Supplement: Supplementary file 1 [file pathophysiology-30-00020-s001.zip › pathophysiology-2318784-supplementary.pdf]

Study or Subgroup	≥65 years old Events	Total	<65 years old Events	Total	Weight	Odds Ratio M-H, Random, 95% CI
Naaraayan 2020	129	211	53	120	56.4%	1.99 [1.26, 3.13]
Vee 2020	3	5	13	242	43.6%	26.42 [4.05, 172.20]
**Total (95% CI)**		**216**		**362**	**100.0%**	**6.15 [0.49, 76.62]**
Total events	132		66			

Heterogeneity: Tau² = 2.89; Chi² = 6.96, df = 1 (P = 0.008); I² = 86%
Test for overall effect: Z = 1.41 (P = 0.16)

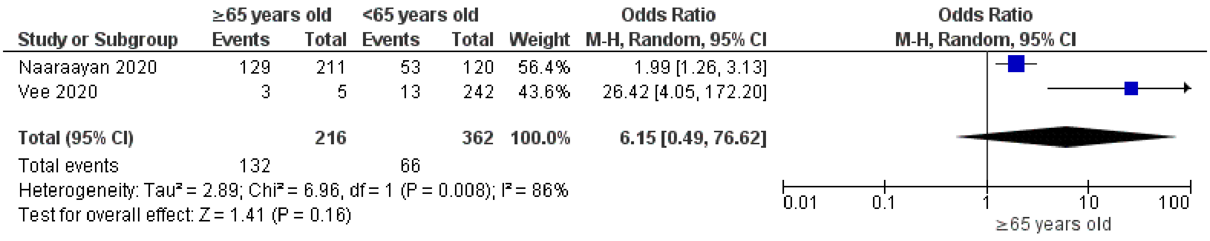

(a)

Study or Subgroup	Obesity Events	Total	No Obesity Events	Total	Weight	Odds Ratio M-H, Random, 95% CI
Fisher 2020	726	1351	1069	1797	27.0%	0.79 [0.69, 0.91]
Fominskiy 2020	16	22	56	74	5.1%	0.86 [0.29, 2.52]
Hirsch 2020	559	1475	1434	3974	27.5%	1.08 [0.96, 1.22]
Joseph 2020	18	21	63	79	3.5%	1.52 [0.40, 5.82]
Lee 2020	71	184	223	818	20.1%	1.68 [1.20, 2.34]
Nakeshbandi 2020	33	215	62	289	15.6%	0.66 [0.42, 1.06]
Taher 2020	2	3	27	70	1.2%	3.19 [0.28, 36.85]
**Total (95% CI)**		**3271**		**7101**	**100.0%**	**1.02 [0.78, 1.33]**
Total events	1425		2934			

Heterogeneity: Tau² = 0.06; Chi² = 25.05, df = 6 (P = 0.0003); I² = 76%
Test for overall effect: Z = 0.14 (P = 0.89)

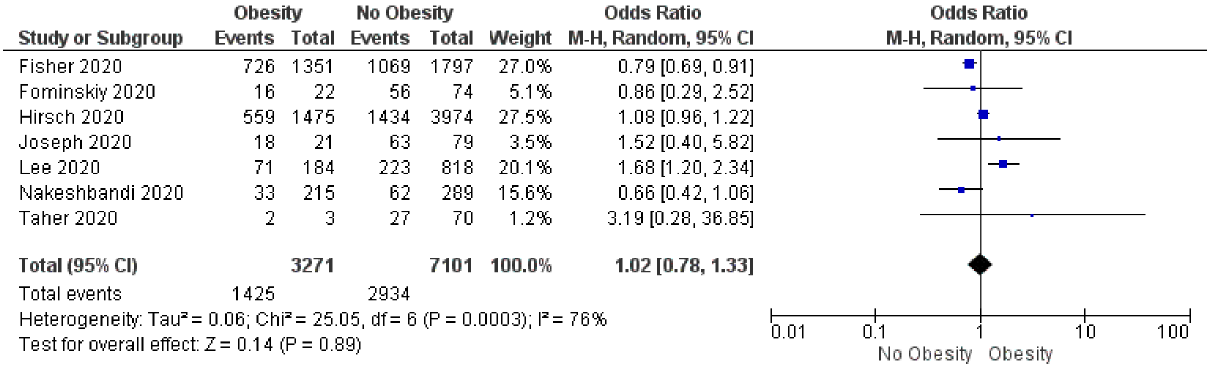

(b)

Study or Subgroup	Smokers Events	Total	non-Smokers Events	Total	Weight	Odds Ratio M-H, Fixed, 95% CI
Dudoignon 2020	4	6	22	45	4.6%	2.09 [0.35, 12.59]
Fominskiy 2020	3	5	69	91	7.7%	0.48 [0.08, 3.05]
Naar 2020	35	47	113	159	35.3%	1.19 [0.57, 2.49]
Xia 2020	7	12	34	69	11.2%	1.44 [0.42, 4.98]
Zahid 2020	8	38	120	431	41.1%	0.69 [0.31, 1.55]
**Total (95% CI)**		**108**		**795**	**100.0%**	**1.00 [0.63, 1.57]**
Total events	57		358			

Heterogeneity: Chi² = 2.60, df = 4 (P = 0.63); I² = 0%
Test for overall effect: Z = 0.00 (P = 1.00)

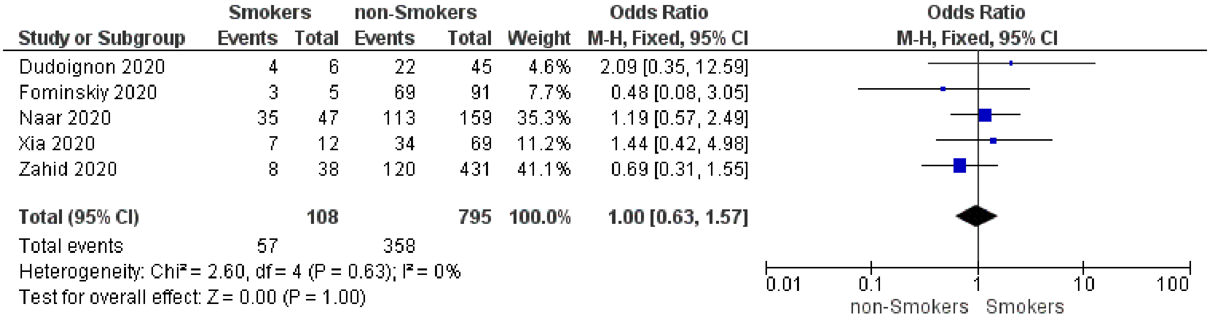

(c)

Study or Subgroup	Cerebrovasc. disease Events	Total	No cerebrovasc. disease Events	Total	Weight	Odds Ratio M-H, Random, 95% CI
Russo 2020	44	128	132	649	35.2%	2.05 [1.36, 3.10]
Xia 2020	2	11	39	70	12.3%	0.18 [0.04, 0.88]
Yan 2020	7	38	108	844	24.8%	1.54 [0.66, 3.58]
Zahid 2020	12	37	116	432	27.7%	1.31 [0.64, 2.69]
**Total (95% CI)**		**214**		**1995**	**100.0%**	**1.25 [0.63, 2.45]**
Total events	65		395			

Heterogeneity: Tau² = 0.29; Chi² = 9.08, df = 3 (P = 0.03); I² = 67%
Test for overall effect: Z = 0.64 (P = 0.52)

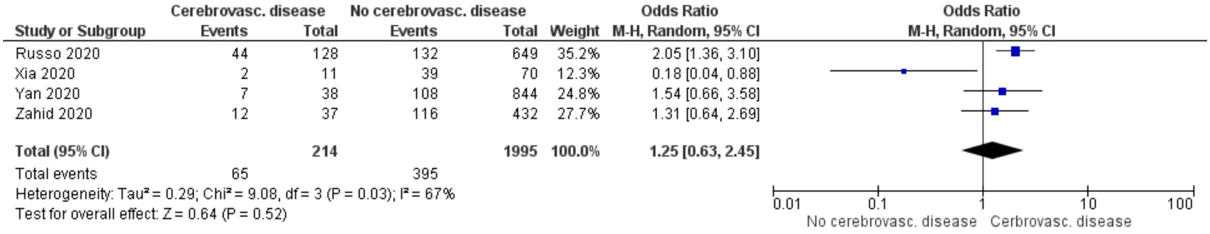

(d)

Study or Subgroup	Cancer Events	Cancer Total	No cancer Events	No cancer Total	Weight	Odds Ratio M-H, Fixed, 95% CI
Hirsch 2020	133	327	1860	5122	88.3%	1.20 [0.96, 1.51]
Lim 2020	7	16	23	144	1.7%	4.09 [1.38, 12.09]
Taher 2020	3	5	26	68	1.0%	2.42 [0.38, 15.49]
Wang 2020	1	10	11	106	1.1%	0.96 [0.11, 8.31]
Zahid 2020	8	31	120	438	7.9%	0.92 [0.40, 2.12]
**Total (95% CI)**		**389**		**5878**	**100.0%**	**1.24 [1.00, 1.53]**
Total events	152		2040			

Heterogeneity: Chi² = 5.78, df = 4 (P = 0.22); I² = 31%
Test for overall effect: Z = 1.98 (P = 0.05)

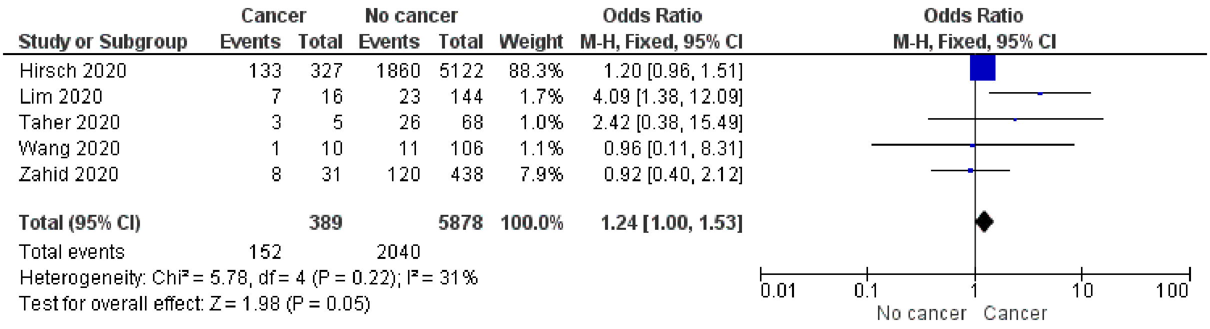

(e)

Study or Subgroup	Chronic liver disease Events	Chronic liver disease Total	No Chronic liver disease Events	No Chronic liver disease Total	Weight	Odds Ratio M-H, Fixed, 95% CI
Chan 2020	99	191	1736	3802	60.8%	1.28 [0.96, 1.71]
Hirsch 2020	42	114	1951	5335	39.2%	1.01 [0.69, 1.49]
**Total (95% CI)**		**305**		**9137**	**100.0%**	**1.18 [0.93, 1.48]**
Total events	141		3687			

Heterogeneity: Chi² = 0.92, df = 1 (P = 0.34); I² = 0%
Test for overall effect: Z = 1.37 (P = 0.17)

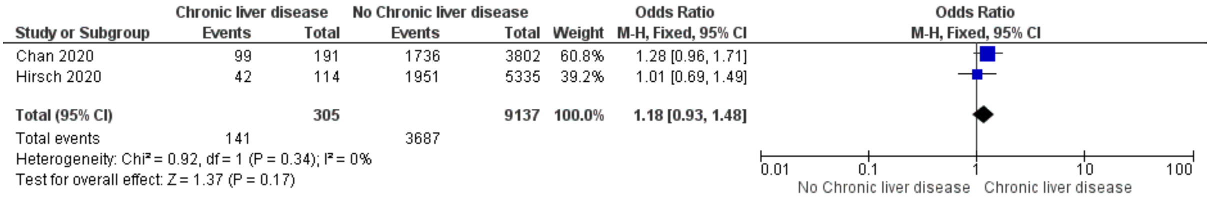

(f)

Study or Subgroup	HIV Events	HIV Total	No HIV Events	No HIV Total	Weight	Odds Ratio M-H, Fixed, 95% CI
Hirsch 2020	10	35	1983	5414	78.0%	0.69 [0.33, 1.44]
Pelayo 2020	4	6	106	217	8.2%	2.09 [0.38, 11.67]
Zahid 2020	2	8	126	461	13.8%	0.89 [0.18, 4.45]
**Total (95% CI)**		**49**		**6092**	**100.0%**	**0.83 [0.45, 1.53]**
Total events	16		2215			

Heterogeneity: Chi² = 1.36, df = 2 (P = 0.51); I² = 0%
Test for overall effect: Z = 0.59 (P = 0.56)

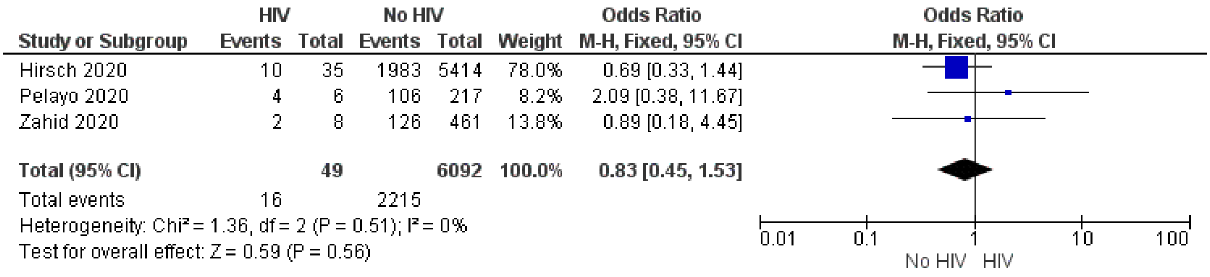

(g)

Study or Subgroup	Aspirin use Events	Aspirin use Total	No Aspirin use Events	No Aspirin use Total	Weight	Odds Ratio M-H, Fixed, 95% CI
Fominskiy 2020	14	15	58	81	3.6%	5.55 [0.69, 44.68]
Zahid 2020	41	128	87	341	96.4%	1.38 [0.88, 2.14]
**Total (95% CI)**		**143**		**422**	**100.0%**	**1.53 [1.00, 2.34]**
Total events	55		145			

Heterogeneity: Chi² = 1.68, df = 1 (P = 0.19); I² = 41%
Test for overall effect: Z = 1.95 (P = 0.05)

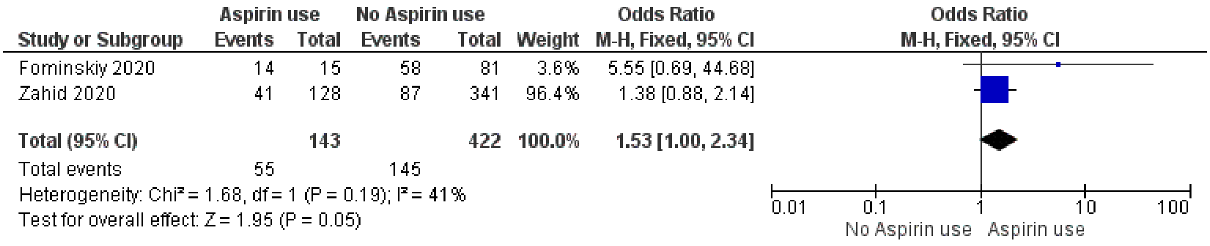

(h)

Figure S1. Forrest plot showing association between risk of AKI in COVID-19 patients with advanced age (a), obesity (b), smokers (c), cerebrovascular disease (d), cancer (e), chronic liver disease (f), HIV (g), and chronic use of aspirin (h). [11,14-16,18,20,21,23,26,28,30,31,33-35,37-39]
